# Efficacy of casein phosphopeptide-amorphous calcium phosphate to prevent stain absorption on freshly bleached enamel: An *in vitro* study

**DOI:** 10.4103/0972-0707.66715

**Published:** 2010

**Authors:** Raghuwar D Singh, Sabita M Ram, Omkar Shetty, Pooran Chand, Rakesh Yadav

**Affiliations:** Department of Prosthodontics, Faculty of Dental Sciences, C.S.M. Medical University, Lucknow, Uttar Pradesh, India; 1 Department of Prosthodontics, Padm. Dr. D.Y. Patil Dental College and Hospital, Nerul, Navi Mumbai, Maharashtra, India; 2 Department of Conservative Dentistry, Faculty of Dental Sciences, C.S.M. Medical University, Lucknow, Uttar Pradesh, India

**Keywords:** Bleaching, fluoride, casein phosphopeptide – amorphous calcium phosphate, stain absorption, spectrophotometer

## Abstract

**Background:**

Teeth when subjected to bleaching bring about the desiccation of the enamel, making it more susceptible to stain absorption. While subjecting the freshly bleached enamel surface to various surface treatments of Fluoride and Casein Phosphopeptide - Amorphous Calcium phosphate (CPP-ACP) brought about the reduction in stain absorption, which is assessed in this study.

**Aims:**

The study aims to evaluate the tea stain absorption on freshly bleached enamel surface of extracted human teeth with varied surface treatment. The stain absorption was evaluated at the end of one hour and 24 hours post bleaching.

**Materials and Methods:**

Forty extracted human permanent maxillary central incisors were subjected to bleaching with 10% carbamide peroxide for eight days. They were divided into four groups of 10 each. Group I was control group. Group II was immersed in tea solution without surface treatment, while Group III and IV were immersed in tea solution with surface treatment of topical Fluoride and CPP-ACP respectively. Spectrophotometer was used for color analysis.

**Results:**

Surface treatment with CPP-ACP and topical fluoride on freshly beached enamel surface, significantly reduced the stain absorption.

**Conclusion:**

Remineralizing agents reduce stain absorption after tooth bleaching.

## INTRODUCTION

Vital tooth bleaching with Carbamide Peroxide (CP) gels is becoming more and more popular. Although no macroscopically, clinically remarkable damages because of vital bleaching of the dental hard tissues have been described in literature, there are scientific reports which demonstrate alterations of the histological aspects and composition of bleached dental enamel.[1] It is observed that bleaching with 10% CP may result in a decrease of the calcium and phosphate content and also of the fluoride amount in enamel.[2] In a previous study, it was shown that the loss of micro hardness in bleached enamel could be outweighed by a remineralization period following the bleaching period.[3,4] It may be speculated that in this case micro structural defects may be repaired by the absorption and precipitation of components of the saliva, such as calcium and phosphate. Casein phosphopeptide-amorphous calcium phosphate (CPP-ACP) has been shown to prevent enamel demineralization and promote remineralization of enamel surface lesions in animal and human *in situ* caries models.

It is conceivable that a dietary component, such as tea consumed during or just after the completion of bleaching treatment may lead to staining of the bleached enamel surface.[5,6] It is further unknown whether an appropriate length of remineralization time should elapse after bleaching, and before consumption of tea. Although numerous studies on bleaching have been reported, little research is available to evaluate the effect of tea, coffee, cola etc. on the color of freshly bleached enamel surface of human teeth. The literature review also does not show much study done to analyze the effect of various remineralizing agents such as Fluoride and CPP-ACP applied over the freshly bleached enamel surface of human teeth, to prevent stain absorption.

It was thought if the enamel of freshly bleached tooth was surface treated, so that along with the effect of reduced sensitivity, it may reduce the absorption of stains and therefore maintain the effect of bleaching for a longer time.[7] Hence, an attempt was made to evaluate the freshly bleached enamel surface of extracted human teeth for stain absorption subjected to varied surface treatment.

## MATERIALS AND METHODS

### Sample size

Forty extracted human permanent maxillary central incisors were selected for the study. It was seen that the selected teeth were free of dental caries or restorations and were of normal crown anatomy. The teeth were stored in 5% normal saline at room temperature from the day of extraction until the testing was done.

Each tooth was numbered on the root portion. For spectrophotometric evaluation, each of the tooth samples was embedded in white colored hard carving wax with approximately 10mm. thickness, to keep the tooth surface flat to the beam of the spectrophotometer. Wax was used so that the samples could be easily retrieved after each spectrophotometric analysis.

### Color evaluation

All the samples were analyzed under the spectrophotometer at the following stages in the study,

- Pre - bleaching evaluation
- Post - bleaching evaluation: -
  - After eight days of bleaching
  - At the end of one hour of bleaching
  - At the end of 24 hours of bleaching

Spectrophotometer used for this study was, “Spectrophotometer (Spectra flash 500) by data color international (Lawrenceville, NJ)”. The aperture of the data-color spectrophotometer used in the study was 3.0mm in diameter. Readings were recorded in CIELAB color system in the form of L*, a*, b*. L* characterizes the lightness and can range between 0 (dark) to 100 (light). The value of a* defines a color on a red - green axis and b* defines a color on a yellow - blue axis. The total change in color (dE*) of all the teeth samples was calculated by using following formula,

$$\mathrm{dE}*\;=\;\sqrt{\;\mathrm{(dL}*)\;2\;+\;\mathrm{(da}*)\;2\;+\mathrm{(db}*){}^{2}}$$

Where dL*, da*, and db* represent the difference in L*, a*, and b* values, respectively.

### Bleaching procedure

All teeth samples were placed in petri dish with the help of an adhesive (Fevi Kwik) to stabilize them in their position. The labial surface of the samples was single coated with 10% Carbamide peroxide bleaching agent (Opalescence - Ultradent products, Inc.) using a brush to have approximately 1mm thickness of the material. This was left in place for 8 hours for bleaching of the samples. After eight hours of bleaching period, the samples were stored in artificial saliva for the next 16 hours and again subjected to the bleaching process. This cycle was continued for eight successive days. Freshly prepared artificial saliva was used for storage of teeth during bleaching period.[8]

### Surface treatments

After bleaching, all 40 teeth samples were divided into four groups, each group containing ten teeth samples. Surface treatment was done with group III and IV.

Group I - Samples were stored in artificial saliva without placing in tea solution. Group II - Samples were stored in artificial saliva after placing in tea solution, but without surface treatment. Group III - Fluoride gel (Fluorovil - Topical Fluoride Thixotropic gel, acidulated phosphate fluoride 1.23%) was applied with a brush, over the dried labial surface of each of the samples and left for five minutes. Group IV - Casein Phosphopeptide - Amorphous Calcium phosphate (CPP-ACP; GC Tooth mousse remineralizing agent – Ultradent Products Inc.) was applied with a brush, over the dried labial surface of the samples and left for five minutes.

After five minutes, gel over the enamel surface of group III and IV was gently wiped off with gauze and the teeth samples were stored in artificial saliva. After one hour, group II, III and IV were removed from the artificial saliva and immersed in freshly prepared tea solution for 10 minutes. The tea solution was prepared by boiling two grams of tea (Girnar CTC tea; Girnar Food and Beverages Pvt Ltd., Kurla, Mumbai, India) in 100ml. of distilled water for 5 minutes, and filtered through a strainer. Fresh tea solution was made each day for the study.

After 10 minutes, samples were removed from the tea solution, washed thoroughly, dried with gauze, mounted on wax blocks and color of each of the sample was evaluated. After which the samples were restored in artificial saliva for next 24 hours. At the end of 24 hours, the above samples were again removed from the artificial saliva, immersed in freshly prepared tea solution for 10 minutes and evaluated by using spectrophotometer.

## RESULTS

The results were analyzed statistically by Students ‘t’ test. Total color change (dE*) of all Groups I to IV is shown in Tables 1 and 2. Result showed that all the teeth samples when bleached with 10% CP for a period of eight days became lighter, showing a significant change in color.

**Table 1:** Mean changes in values L*a*b* and total color change dE* of all groups, after immersing group II, III and IV in tea solution at the end of 1 hour after bleaching

Groups		dL*	da*	db*	dE*
I	Mean	- 0.21	0	+ 0.01	0.21
	SD	(± 2.31)	(± 1.24)	(± 0.68)	
	*P*-value	*P* > 0.05	*P* > 0.05	*P* > 0.05	
		NS	NS	NS	
II	Mean	3.23	0.65	1.49	3.97
	SD	(± 1.20)	(± 0.44)	(± 1.54)	
	*P*-value	*P* < 0.05	*P* > 0.05	*P* > 0.05	
		S	NS	NS	
III	Mean	2.63	0.29	0.22	2.74
	SD	(± 0.31)	(±0.65)	(± 2.21)	
	*P*-value	*P* < 0.05	*P* > 0.05	*P* > 0.05	
		S	NS	NS	
IV	Mean	-2.2	0.356	0.58	2.54
	SD	(± 0.61)	(± 0.48)	(± 2.83)	
	*P*-value	*P* > 0.05	*P* > 0.05	*P* > 0.05	
		NS	NS	NS	

SD = Standard deviation, NS = Statistically non significant, S = Statistically significant

**Table 2:** Mean changes in values L*a*b* and total Color change dE* of all groups, after immersing group II, III and IV in tea solution at the end of 24 hour after bleaching

Groups		dL*	da*	db*	dE*
I	Mean	- 0.08	0.098	0.04	0.19
	SD	(± 1.67)	(±0.59)	(±1.17)	
	*P*-value	*P* > 0.05	*P* > 0.05	*P* > 0.05	
		NS	NS	NS	
II	Mean	-1.30	0.404	2.57	4.64
	SD	(± 4.27)	(± 0.63)	(± 2.62)	
	*P*-value	*P* < 0.05	*P* > 0.05	*P* > 0.05	
		S	NS	NS	
III	Mean	-3.48	0.312	0.61	3.50
	SD	(± 1.61)	(± 0.61)	(± 1.93)	
	*P*-value	*P* < 0.05	*P* > 0.05	*P* > 0.05	
		S	NS	NS	
IV	Mean	-2.2	0.466	0.58	3.16
	SD	(± 0.61)	(± 0.42)	(± 2.63)	
	*P*-value	*P* > 0.05	*P* > 0.05	*P* > 0.05	
		NS	NS	NS	

SD = Standard deviation, NS = Statistically non significant, S = Statistically significant

Group I samples showed a very slight change in dL* value towards the negative (darker) direction at the end of 1 hour (dE*=0.21) after bleaching. The change in total color dE* was almost same at the end of 24 hours (dE*=0.19) after bleaching as shown in Tables 1 and 2. Group II samples, at the end of 1 hour after bleaching, showed a significant change in dE* value due to decrease in dL* (darker) value. The color of the sample shifted towards the darker side. There was little change in the da* and db* value towards the positive direction. The change of dL* value in negative direction represents that the teeth samples of group II absorbed tea stain and become darker in color. There was also a significant change in total color dE*, when the group II samples were immersed in tea solution at the end of 24 hours. This change was due to change in dL* value towards the negative (darker) direction, which was statistically significant. This indicated that even at the end of 24 hours after bleaching, the bleached tooth surface absorbed the tea stain. Group III and group IV both showed the change in total color (dE*), at the end of one hour and 24 hours as shown in Tables 1 and 2. The change was due to the shift in dL* value towards the negative (darker) direction.

Result showed that stain absorption gradually increased from one to 24 hours as shown in Tables 1 and 2, but the rate of absorption reduced. Rate of stain absorption was greater at the end of 1 hour after bleaching.

## DISCUSSION

Bleaching restores normal color to a tooth by decolorization of stain with a powerful oxidizing or reducing agent. Attin on bovine teeth showed that application of tea solution directly after bleaching with 10% CP, changes the color of enamel towards the darker side.[6] He also advocated the application of fluoride over the bleached enamel surface; to improve the remineralization to reduce sensitivity.[4] CPP-ACP enhances remineralization of enamel surfaces by occluding the dentinal tubules, which results in reduced hypersensitivity.[7,9]

Eight-hour period of bleaching was followed by placing the samples in freshly prepared artificial saliva for 16 hours. Storage in saliva was chosen, to simulate the oral environment. Freshly prepared artificial saliva used in the study, represents the kind of average saliva with respect to organic and inorganic components, normally found in human saliva. The saliva was mixed daily according to Klimek, Helluig and Ahrens to get the fresh saliva.[8] Furthermore, human saliva from different individuals has varying properties, and also the pH of saliva differs from person to person. Therefore, the artificial saliva was used instead of human saliva in order to standardize the conditions in the study.

The eight-hour period of bleaching was chosen to simulate the condition of wearing bleaching tray overnight.[6] Fluoride gel and CPP-ACP were used in the study for surface treatment, just after bleaching procedure. Fluoride gel and CPP-ACP enhance the remineralization of enamel surface, which had been demineralized after bleaching.[8]

The storage in tea for 10 minutes surely represents a long period as compared with the shorter contact of the teeth during tea intake. We intended to expose the samples for 10 minutes to get the maximum effect of stain. The reason to use tea as a solution for staining of the teeth was, that tea was proved to have a higher capacity to stain teeth than other staining solutions.[5]

Bleaching caused a color shift towards the blue direction within the color space and lightened the color of the teeth. The observed tooth color change was mainly due to a shift of the L* value towards the positive side (more brightness) and the b* value towards the negative side (less yellow and more blue). The a* value also decreased (less red and more green) after bleaching, but it had only a minor influence on the total tooth color change (dE*).

It was observed that groups II, III, and IV showed the greater change in color (dE*) after immersing in tea solution at the end of one hour, than those of 24 hours. This could be due to the remineralizing effect of artificial saliva, Fluoride, and CPP-ACP solution, which tend to prevent the stain absorption more effectively at the end of 24 hours after surface treatment.

Out of the various surface treatments, CPP-ACP (group IV) and Fluoride (group III) showed a reduced total color change after immersing in tea solution, compared to without surface treated teeth samples (group II). These findings were due to the remineralizing capacity of CPP-ACP and Fluoride. In the earlier studies it has been demonstrated that CPP-ACP reduces the hypersensitivity by occluding the dentinal tubules.[7] Fluoride improved the remineralization, when applied on the bleached enamel surface. Hence, this study showed that CPP-ACP and Fluoride not only reduce the sensitivity, but also reduce the stain absorption.

Within the limitations of this *in vitro* study, the following conclusions were drawn:

1. Teeth when stored in artificial saliva with no surface treatment and without subjecting to stain in tea solution did not show any significant change in color. This could be due to the remineralizing effect of artificial saliva.
2. Stain absorption was found to be least when tooth was surface treated with CPP-ACP.
3. Stain absorption was reduced and tooth surfaces showed more color stability with surface treatment of remineralizing agents.
4. This study provides a solution to reduce stain absorption by the application of CPP-ACP and fluoride, which along with reducing sensitivity would also reduce the absorption of stain in the period immediately after bleaching, when the surface is still demineralized.

## References

[1] McGuckin RS, Babin JF, Meyer BJ (1992). Alternation in human enamel surfaces morphology following vital bleaching. J Prosthet Dent.

[2] Potocnik I, Kosec L, Gaspersic D (2000). Effect of 10% carbamide peroxide bleaching gel on enamel microhardness, microstructure, and mineral content. J Endod.

[3] Shannon H, Spencer P, Gross K, Tira D (1993). Characterization of enamel exposed to 10% Carbamide peroxide bleaching agents. Quintessence Int.

[4] Attin T, Kielbarsa AM, Schwanenberg M, Hellwig E (1997). Effect of fluoride treatment on reminerelization of bleached enamel. J Oral Rehabil.

[5] Leard A, Addy M (1997). Propensity of different brands of Tea and Coffee to cause staining associated with chlorhexidine. J Clin Perio.

[6] Attin T, Manolakis A, Buchalla W, Hannig C (2003). Influence of tea on intrinsic colour of previously bleached enamel. J Oral Rehabil.

[7] Suge T, Ishikawa K, Kawasaki A, Yoshiyama M, Asaoka K, Ebisu S (1995). Effect of Fluoride on the calcium phosphate precipitation method for dentinal tubules occlusion. J Dent Res.

[8] Klimek J, Hellwig E, Ahrens G (1982). Fluoride taken up by plaque, by the underlying enamel and by the clean enamel from three Fluoride compounds *in vitro*. Caries Res.

[9] Cai F, Shan P, Morgan MV, Reynolds EC (2003). Free lozenges containing casein phosphopeptide - amorphous calcium phosphate (CPP-ACP). Aust Dent J.

